# *In Silico* Analysis of Differential Gene Expression in Three Common Rat Models of Diastolic Dysfunction

**DOI:** 10.3389/fcvm.2018.00011

**Published:** 2018-02-21

**Authors:** Raffaele Altara, Fouad A. Zouein, Rita Dias Brandão, Saeed N. Bajestani, Alessandro Cataliotti, George W. Booz

**Affiliations:** ^1^Institute for Experimental Medical Research, Oslo University Hospital and University of Oslo, Oslo, Norway; ^2^KG Jebsen Center for Cardiac Research, Oslo, Norway; ^3^Department of Pathology, School of Medicine, University of Mississippi Medical Center, Jackson, MS, United States; ^4^Faculty of Medicine, Department of Pharmacology and Toxicology, American University of Beirut, Beirut, Lebanon; ^5^Department of Clinical Genetics, Maastricht University Medical Centre, Maastricht, Netherlands; ^6^Department of Ophthalmology, School of Medicine, University of Mississippi Medical Center, Jackson, MS, United States; ^7^Department of Pharmacology and Toxicology, School of Medicine, University of Mississippi Medical Center, Jackson, MS, United States

**Keywords:** metabolic disease, endothelial and microvascular dysfunction, inflammation, heart failure, hypertension

## Abstract

Standard therapies for heart failure with preserved ejection fraction (HFpEF) have been unsuccessful, demonstrating that the contribution of the underlying diastolic dysfunction pathophysiology differs from that of systolic dysfunction in heart failure and currently is far from being understood. Complicating the investigation of HFpEF is the contribution of several comorbidities. Here, we selected three established rat models of diastolic dysfunction defined by three major risk factors associated with HFpEF and researched their commonalities and differences. The top differentially expressed genes in the left ventricle of Dahl salt sensitive (Dahl/SS), spontaneous hypertensive heart failure (SHHF), and diabetes 1 induced HFpEF models were derived from published data in Gene Expression Omnibus and used for a comprehensive interpretation of the underlying pathophysiological context of each model. The diversity of the underlying transcriptomic of the heart of each model is clearly observed by the different panel of top regulated genes: the diabetic model has 20 genes in common with the Dahl/SS and 15 with the SHHF models. Advanced analytics performed in Ingenuity Pathway Analysis (IPA^®^) revealed that Dahl/SS heart tissue transcripts triggered by upstream regulators lead to dilated cardiomyopathy, hypertrophy of heart, arrhythmia, and failure of heart. In the heart of SHHF, a total of 26 genes were closely linked to cardiovascular disease including cardiotoxicity, pericarditis, ST-elevated myocardial infarction, and dilated cardiomyopathy. IPA Upstream Regulator analyses revealed that protection of cardiomyocytes is hampered by inhibition of the ERBB2 plasma membrane-bound receptor tyrosine kinases. Cardioprotective markers such as natriuretic peptide A (*NPPA*), heat shock 27 kDa protein 1 (*HSPB1*), and angiogenin (*ANG*) were upregulated in the diabetes 1 induced model; however, the model showed a different underlying mechanism with a majority of the regulated genes involved in metabolic disorders. In conclusion, our findings suggest that multiple mechanisms may contribute to diastolic dysfunction and HFpEF, and thus drug therapies may need to be guided more by phenotypic characteristics of the cardiac remodeling events than by the underlying molecular processes.

## Introduction

Comparatively isolated diastolic dysfunction or stiffness of the left ventricle is a chronic pathological condition that evolves into heart failure with preserved ejection fraction (HFpEF). An increasing body of evidence reports that HFpEF is frequent in women and the elderly (1–3). In fact, HFpEF accounts for about half the cases of heart failure (HF) and is the leading cause of hospital admission in patients over 65 years of age. Although individuals with HFpEF exhibit similar mortality rates as those that have heart failure with reduced ejection fraction (HFrEF) or impaired cardiac contractility, there are currently no proven effective medicines for this condition (1, 4–6) Thus, HFpEF is a major unmet medical need and there is an urgency for new therapeutic approaches and strategies that target mechanisms specific for HFpEF. Notably, HFpEF is associated with the co-presence of several comorbidities (7). Particularly among younger persons, HFpEF is associated with the interrelated cardiovascular risk factors of obesity and type II diabetes (8). Given the obesity epidemic that is attributed to the Western diet and lifestyle, and an aging population, it is not surprising then that HFpEF is predicted to be the primary cause of HF within a decade.

A clinical cardiac phenotype like HFpEF is difficult to study due to its complex nature that involves genetic, molecular, and environmental factors. Ultimately, increased cardiac stiffness in HFpEF is thought to arise from loss of nitric oxide signaling due to microvascular inflammation, increased cardiac fibrosis, and a concentric pattern of cardiac hypertrophy that also compromises ventricular chamber size. Several animal models are characterized by diastolic dysfunction and have been used to study HFpEF. However, it is unclear what these different models have in common, or their differences, as far as disease etiology and cardiac remodeling, which could reveal common or fundamental signaling pathways representing potential therapeutic targets. Given the rapid growth of experimental genetic data in repositories, such as Gene Expression Omnibus (GEO), data mining is a powerful approach that can create countless openings for holistic investigations using assorted studies.

Our *in silico* investigation aimed towards identifying novel pathological pathways in the heart that are relevant for the development of HFpEF. In our study, we make use of public datasets, to perform top-notch biostatistics and bioinformatics that aid in deciphering genetic events that drive the detrimental cardiac remodeling in three rodent models with diastolic dysfunction, the Dahl salt sensitive rat (Dahl/SS), the spontaneously hypertensive heart failure (SHHF) rat, and the type I diabetic rat.

## Methods

### GEO Search

GEO search for microarray data from heart in three animal models with underlying diastolic disfunction: the Dahl Salt Sensitive (GSE66617), the Spontaneous Hypertensive Heart Failure (GSE2876), and the Streptozotocin/Diabetes1 (GSE6880). The total number of samples of each dataset was *n* = 28, 10 and 6 for Dahl SS, SHHF, and diabetic, respectively.

### Rat Models Selected

Dahl Salt Sensitive (SS) rats were fed either a 0.3% NaCl (normal salt, NS, control group) or with 8% NaCl (high salt, HS, heart failure group) diet until evidence of left ventricular dysfunction (9). The authors extracted total RNA from left ventricular samples at the stage of heart failure using mirVana (Applied Biosystems) according to the manufacturer’s instructions (9). Three samples from each group were pooled. cDNA and Amino Allyl aRNA was synthesized by Amino Allyl MessageAmp II aRNA Amplification Kit (Ambion). CyeDye Coupling and fragmentation were performed according to the manufacturer’s protocol (TORAY Industries, Inc, Tokyo, Japan). Samples were then hybridized for 16 h at 37°C on a Rat Oligo chip 20 K (TORAY Industries, Inc, Tokyo, Japan). Scanning was performed on a 3D-Gene Scanner (Toray Industries Inc., Tokyo, Japan).

Spontaneously Hypertensive and Heart Failure-prone (SHHF) rats were obtained by backcrossing a Koletsky obese rat to a Spontaneously Hypertensive Rat (SHR/N) (10). Controls were 4 months old lean rats and heart failure rats were 10 months old obese rats (10). Total RNA was extracted with TRIzol (GIBCO) according to the manufacturer’s protocol and reverse transcribed with Superscript enzyme (Invitrogen) and labelling the cDNA with ^33^P. Purification of cDNA was performed using NucleoSpin Extract (Macherey-Nagel). The kit Array-Advantage GF (Ambion) was used to hybridize the samples into pan-genomic macroarray nylon membranes (RZPD, Berlin), which were exposed for 24 h and scanned with a Scan Typhoon 9400 (resolution at 50 µm per pixel).

Diabetic rats were induced with streptozotocin injection at 8 weeks, which led to a model of insulin-deficient type 1 diabetes (11). Rats were sacrificed at 12 weeks (so diabetic for 4 weeks) (11). RNA was extracted with TRIzol (GIBCO) and cleaned with RNeasy total RNA mini kit (Qiagen). Superscript II (Life Technologies) was used for first strand cDNA synthesis. Subsequent steps necessary to hybridize the samples on Affymetrix RAE 230A microarrays were performed according to the Affymetrix protocol. Arrays were scanned on an Hewlett-Packard Gene Array scanner.

### Microarray Data Analysis

GEO2R has been used as a base for the analysis of the Dahl/SS dataset, whereas the Limma package was used to analyze the Diabetic and SHHF model. Distribution of value data for the Samples used was calculated (data not shown) and median-centered values indicated that the data were normalized and cross-comparable. Fold-changes per group were calculated using average Disease/Control. Statistical significance of differences in gene expression was assessed using the nonparametric Wilcoxon signed-rank test and the log2-transformed data.

The differentially expressed genes (DEGs) with fold-change (FC) >2 and < −2 and adjusted *p*-value < 0.05 have been selected for Dahl/SS and SHHF, whereas FC >1.5 and FC < −1.5 was chosen for Diabetic 1 due to the high level of confidence [false discovery rate (FDR) = 0, (Figure S1)] at *p*-values < 0.05.

The Benjamini and Hochberg false discovery rate method was selected by default because it is the most commonly used adjustment for microarray data and provides a good balance between discovery of statistically significant genes and limitation of false positives.

Gene Set Enrichment Analysis (GSEA) was performed for each of the three datasets using QIAGEN’s Ingenuity Pathway Core Analysis (IPA, QIAGEN Redwood City, www.qiagen.com/ingenuity). The Ingenuity Knowledge Base was used as reference set and both direct and indirect experimentally confirmed relationships from humans and rodents. Z-scores of >2 or < −2 were considered significant. Details about the statistics used can be found at the Ingenuity website: http://www.ingenuity.com/products/ipa#/?tab=resources.

Genes have been mapped in IPA for further analysis (see Table 1).

**Table 1:** DEGs analysed in IPA

**GEO Series (GSE)**	**Rat Model**	**Condition**	**Cases**	**Controls**	**DEGs**		**IPA Mapped IDs**	**IPA Unmapped IDs**
					**Increased**	**Decreased**		
GSE66617	Dahl/SS	Systemic Hypertension	3	3	222	28	80	53
GSE2876	SHHF	Hypertension,Obesity	17	11	81	114	172	8
GSE6880	Streptozotocin (Diabetes1)	Diabetes,Obesity	3	3	593	546	1,062	62

## Results

### Animal Models of HFpEF

Our GEO search for published microarray data on hearts from animal models of diastolic dysfunction was narrowed down to the Dahl/SS, streptozotocin-induced diabetic, and SHHF rat models. Table 1 shows the overall number of DEGs found for each animal model. The top 250 DEGs (genes with the smallest *p*-value) calculated with GEO2R indicated that the diabetic model has 20 genes in common with the Dahl/SS and 15 with the SHHF models (Table 2). Given the high cumulative number of significant calls for the diabetic rat model (Figure S1) we included up to 1,139 genes with a local FDR (false discovery rate) of 0.

**Table 2:** Common DEGs among the HRpEF animal models

**Animal models**	**Gene names**	**Entrez Gene Name**	**Location**	**Type**
**Dahl/SS and SHHF**	ADRA1D	Adrenoceptor alpha 1D	Plasma Membrane	G-protein coupled receptor
	AKIP1	A-kinase interacting protein 1	Nucleus	Other
	ALOX15	Arachidonate 15-lipoxygenase	Cytoplasm	Enzyme
	ANKRD23	Ankyrin repeat domain 23	Nucleus	Other
	APLN	Apelin	Extracellular Space	Other
	Bex1/LOC100912195	Brain expressed, X-linked 1	Cytoplasm	Other
	CDC25B	Cell division cycle 25B	Nucleus	Phosphatase
	COL8A1	Collagen type VIII alpha 1 chain	Extracellular Space	Other
	EPN3	Epsin 3	Cytoplasm	Other
	FHL1	Four and a half LIM domains 1	Cytoplasm	Other
	FIBIN	Fin bud initiation factor homolog (zebrafish)	Cytoplasm	Other
	FXYD3	FXYD domain containing ion transport regulator 3	Plasma Membrane	Other
	Hamp	Hepcidin antimicrobial peptide	Extracellular Space	Other
	MS4A6A	Membrane spanning 4-domains A6A	Other	Other
	MYH6	Myosin heavy chain 6	Cytoplasm	Enzyme
	MYH7	Myosin heavy chain 7	Cytoplasm	Enzyme
	NPPA	Natriuretic peptide A	Extracellular Space	Other
	NUPR1	Nuclear protein 1, transcriptional regulator	Nucleus	Transcription regulator
	SFRP1	Secreted frizzled related protein 1	Plasma Membrane	Transmembrane receptor
	TRDN	Triadin	Cytoplasm	Other
**Diabetes 1 and SHHF**	COL4A1	Collagen type IV alpha 1 chain	Extracellular Space	Other
	COL6A1	Collagen type VI alpha 1 chain	Extracellular Space	Other
	CREG1	Cellular repressor of E1A stimulated genes 1	Nucleus	Transcription regulator
	CXCL3	Chemokine (C-X-C motif) ligand 3	Extracellular Space	Cytokine
	ELK3	ELK3, ETS transcription factor	Nucleus	Transcription regulator
	FAM49B	Family with sequence similarity 49 member B	Extracellular Space	Other
	KANK2	KN motif and ankyrin repeat domains 2	Nucleus	Transcription regulator
	KLF9	Kruppel like factor 9	Nucleus	Transcription regulator
	PIK3IP1	Phosphoinositide-3-kinase interacting protein 1	Cytoplasm	Other
	PLXND1	Plexin D1	Plasma Membrane	Transmembrane receptor
	PTP4A2	Protein tyrosine phosphatase type IVA, member 2	Cytoplasm	Phosphatase
	PTPN3	Protein tyrosine phosphatase, non-receptor type 3	Cytoplasm	Phosphatase
	SIPA1L1	Signal induced proliferation associated 1 like 1	Cytoplasm	Other
	WDR45	WD repeat domain 45	Other	Other
	ZFP532	Zinc finger protein 532	Nucleus	Other

### Cardiac Genes Assessed by GSEA

IPA generated annotations were derived for the DEGs in each of the datasets. Among the three animal models we selected, only the Dahl/SS and SHHF models showed that over-represented genes (*n* = 25 and *n* = 26, respectively) determined by DEG analysis are strongly associated with cardiovascular diseases. The cardiovascular disease phenotypes of these two models appear to have different gene datasets as none of the DEGs identified are in common (Table 3). SHHF with 17 genes is also associated with metabolic disease, whereas the diabetic rat is solely associated with metabolic disease with 175 genes (Table 4). These two datasets appear to have in common phosphoinositide-3-kinase interacting protein 1 (*PIK3IP1*), a negative regulator of PI3K and physiological, but not pathological cardiac hypertrophy (12).

**Table 3:** DEGs linked to cardiovascular disease

**Dahl/SS**				**SHHF**			
**Expr Fold Change**	**Symbol**	**Location**	**Type(s)**	**Expr Log Ratio**	**Symbol**	**Location**	**Type(s)**
10.703	ACMSD	Cytoplasm	Enzyme	–1.55	ACBD3	Cytoplasm	Other
10.591	ADRA1D	Plasma Membrane	G-protein coupled receptor	–0.99	AGER	Plasma Membrane	Transmembrane receptor
12.82	ALAS2	Cytoplasm	Enzyme	–1.237	AOC3	Plasma Membrane	Enzyme
11.761	ALOX15	Cytoplasm	Enzyme	–1.016	ATF6	Cytoplasm	Transcription regulator
11.65	APLN	Extracellular Space	Other	1.474	ATP2B1	Plasma Membrane	Transporter
7.917	COL8A1	Extracellular Space	Other	1.21	C1QTNF5	Plasma Membrane	Transmembrane receptor
8.196	CTGF	Extracellular Space	Growth factor	–1.764	C1S	Extracellular Space	Peptidase
8.809	EGR2	Nucleus	Transcription regulator	1.457	CBFB	Nucleus	Transcription regulator
9.589	FHL1	Cytoplasm	Other	–1.103	COL4A1	Extracellular Space	Other
8.642	FIBIN	Cytoplasm	Other	1.147	COL6A1	Extracellular Space	Other
12.541	GPRC5A	Plasma Membrane	G-protein coupled receptor	–1.194	CXCL16	Extracellular Space	Cytokine
7.862	MYBPC2	Cytoplasm	Other	–1.405	FAM49B	Extracellular Space	Other
10.981	MYH6	Cytoplasm	Enzyme	1.929	KLF9	Nucleus	Transcription regulator
9.756	MYH7	Cytoplasm	Enzyme	2.108	LMOD1	Cytoplasm	Other
11.984	NPPA	Extracellular Space	Other	–1.457	NRARP	Nucleus	Transcription regulator
9.867	PDLIM5	Cytoplasm	Other	–1.421	PARP1	Nucleus	Enzyme
12.151	POSTN	Extracellular Space	Other	1.444	PIK3IP1	Cytoplasm	Other
11.873	RHCE/RHD	Plasma Membrane	Transporter	–1.279	PLXND1	Plasma Membrane	Transmembrane receptor
8.419	S100A8	Cytoplasm	Other	–1.668	POLB	Nucleus	Enzyme
8.976	S100A9	Cytoplasm	Other	–1.381	PRNP	Plasma Membrane	Other
7.527	TGFB2	Extracellular Space	Growth factor	–1.427	PSEN1	Plasma Membrane	Peptidase
12.04	THBS4	Extracellular Space	Other	1.257	PTGS1	Cytoplasm	Enzyme
8.753	TIMP1	Extracellular Space	Cytokine	1.394	SLC6A1	Plasma Membrane	Transporter
11.594	TRDN	Cytoplasm	Other	1.267	SV2A	Cytoplasm	Transporter
12.207	WISP2	Extracellular Space	Growth factor	–2.219	TUBA1C	Cytoplasm	Other
				–1.004	ZNF532	Nucleus	Other

Green indicates the downregulated gene; red indicates the upregulated gene.

**Table 4:** DEGs associated with metabolic disease

**SHHF**				**Diabetes 1**			
Expr Log Ratio	Symbol	Location	Type(s)	Expr Log Ratio	Symbol	Location	Type(s)
–0.99	AGER	Plasma Membrane	Transmembrane receptor	1.136	ABAT	Cytoplasm	Enzyme
–1.454	ARSA	Cytoplasm	Enzyme	–0.602	ABCC8	Plasma Membrane	Transporter
1.534	ARSB	Cytoplasm	Enzyme	0.92	ABCG8	Plasma Membrane	Transporter
–1.764	C1S	Extracellular Space	Peptidase	0.629	ACAA2	Cytoplasm	Enzyme
–1.194	CXCL16	Extracellular Space	Cytokine	–0.776	ACAT2	Cytoplasm	Enzyme
–1.621	DCX	Cytoplasm	Other	–0.697	ADAM10	Plasma Membrane	Peptidase
–1.697	LIG1	Nucleus	Enzyme	–1.219	ADRA1B	Plasma Membrane	G-protein coupled receptor
–1.204	NAT8B	Cytoplasm	Enzyme	–3.176	ADRA1D	Plasma Membrane	G-protein coupled receptor
–1.234	PDK1	Cytoplasm	Kinase	–0.691	AKT1	Cytoplasm	Kinase
1.444	PIK3IP1	Cytoplasm	Other	–1.784	ALDH1A1	Cytoplasm	Enzyme
–1.668	POLB	Nucleus	Enzyme	–1.459	ALDH1L1	Cytoplasm	Enzyme
–1.381	PRNP	Plasma Membrane	Other	2.492	ALOX15	Cytoplasm	Enzyme
–1.427	PSEN1	Plasma Membrane	Peptidase	2.875	AMY2B	Extracellular Space	Enzyme
1.257	PTGS1	Cytoplasm	Enzyme	–1.363	APLN	Extracellular Space	Other
–1.237	SNRNP70	Nucleus	Other	–1.284	APOBEC1	Cytoplasm	Enzyme
1.267	SV2A	Cytoplasm	Transporter	–2.127	ARG1	Cytoplasm	Enzyme
–2.219	TUBA1C	Cytoplasm	Other	–0.711	ATP1A2	Plasma Membrane	Transporter
–1.378	UQCC2	Cytoplasm	Other	2.899	ATP1A4	Plasma Membrane	Transporter
				–1.124	ATP7A	Plasma Membrane	Transporter
				–2.395	AVPR2	Plasma Membrane	G-protein coupled receptor
				1.312	BCL2L1	Cytoplasm	Other
				0.848	BMP2	Extracellular Space	Growth factor
				1.046	BRCA1	Nucleus	Transcription regulator
				–0.927	CA4	Plasma Membrane	Enzyme
				1.356	CACNA1B	Plasma Membrane	Ion channel
				1.014	CACNA1S	Plasma Membrane	Ion channel
				–2.783	CASP8	Nucleus	Peptidase
				0.704	CAT	Cytoplasm	Enzyme
				–0.717	CAV1	Plasma Membrane	Transmembrane receptor
				0.795	CBLB	Nucleus	Enzyme
				0.951	Ccl2	Extracellular Space	Cytokine
				–0.861	CCNB1	Cytoplasm	Kinase
				–1.161	CCND1	Nucleus	Transcription regulator
				–1.261	CCND2	Nucleus	Other
				–1.099	CD44	Plasma Membrane	Other
				0.953	CD55	Plasma Membrane	Other
				–1.914	CDKN2B	Nucleus	Transcription regulator
				0.879	CEBPD	Nucleus	Transcription regulator
				–1.19	CEL	Extracellular Space	Enzyme
				–1.443	CHRNA5	Plasma Membrane	Transmembrane receptor
				2.434	CHRNB1	Plasma Membrane	Transmembrane receptor
				3.049	CNR1	Plasma Membrane	G-protein coupled receptor
				–1.606	COL18A1	Extracellular Space	Other
				–1.411	COL1A1	Extracellular Space	Other
				–1.683	COL1A2	Extracellular Space	Other
				–1.979	COL3A1	Extracellular Space	Other
				–0.757	COL4A1	Extracellular Space	Other
				–0.879	COL4A2	Extracellular Space	Other
				–1.037	COL5A1	Extracellular Space	Other
				–0.883	COL5A2	Extracellular Space	Other
				–1.291	COL5A3	Extracellular Space	Other
				–0.693	COL6A1	Extracellular Space	Other
				–1.028	COL6A3	Extracellular Space	Other
				–1.023	COL8A1	Extracellular Space	Other
				0.586	CPT1A	Cytoplasm	Enzyme
				1.683	CRP	Extracellular Space	Other
				0.823	CST3	Extracellular Space	Other
				–2.373	CTSK	Cytoplasm	Peptidase
				–1.26	CXCL12	Extracellular Space	Cytokine
				–0.965	CYP11A1	Cytoplasm	Enzyme
				2.541	CYP2E1	Cytoplasm	Enzyme
				–1.687	CYP7B1	Cytoplasm	Enzyme
				–0.808	Dclk1	Cytoplasm	Kinase
				1.204	DECR1	Cytoplasm	Enzyme
				0.926	DKK3	Extracellular Space	Cytokine
				–1.807	DPP4	Plasma Membrane	Peptidase
				1.335	DRD4	Plasma Membrane	G-protein coupled receptor
				–0.918	EIF2AK2	Cytoplasm	Kinase
				1.828	EIF4EBP1	Cytoplasm	Translation regulator
				–0.766	EPHA4	Plasma Membrane	Kinase
				1.681	FABP1	Cytoplasm	Transporter
				–0.902	FABP5	Cytoplasm	Transporter
				–1.589	FBN1	Extracellular Space	Other
				–1.834	FBN2	Extracellular Space	Other
				0.68	FDFT1	Cytoplasm	Enzyme
				–0.97	FLNA	Cytoplasm	Other
				–0.801	FYN	Plasma Membrane	Kinase
				3.723	GAL	Extracellular Space	Other
				–1.286	GAP43	Plasma Membrane	Other
				1.627	GCKR	Nucleus	Other
				1.926	GDF15	Extracellular Space	Growth factor
				–0.697	GFM1	Cytoplasm	Translation regulator
				1.799	GLRB	Plasma Membrane	Ion channel
				–1.371	GPD2	Cytoplasm	Enzyme
				1.401	GPNMB	Plasma Membrane	Enzyme
				0.725	GUCY1A3	Cytoplasm	Enzyme
				0.855	HADHA	Cytoplasm	Enzyme
				0.593	HADHB	Cytoplasm	Enzyme
				1.203	HBG2	Cytoplasm	Other
				–1.033	HK2	Cytoplasm	Kinase
				0.846	HMGCL	Cytoplasm	Enzyme
				–0.703	HMGCR	Cytoplasm	Enzyme
				3.441	HMGCS2	Cytoplasm	Enzyme
				–1.702	HOMER1	Plasma Membrane	Other
				1.727	HP	Extracellular Space	Peptidase
				–1.439	HRH1	Plasma Membrane	G-protein coupled receptor
				3.249	HSPA1L	Cytoplasm	Other
				–0.714	HSPB1	Cytoplasm	Other
				1.882	HTR1D	Plasma Membrane	G-protein coupled receptor
				0.894	ID1	Nucleus	Transcription regulator
				–0.657	IGF1	Extracellular Space	Growth factor
				0.675	IGF1R	Plasma Membrane	Transmembrane receptor
				1.624	IGFBP3	Extracellular Space	Other
				–2.072	IGFBP5	Extracellular Space	Other
				0.664	IL1R1	Plasma Membrane	Transmembrane receptor
				1.38	INSIG1	Cytoplasm	Other
				0.95	INSR	Plasma Membrane	Kinase
				–1.237	KCNAB3	Plasma Membrane	Ion channel
				–0.611	KCNJ11	Plasma Membrane	Ion channel
				0.764	KCNK3	Plasma Membrane	Ion channel
				1.819	KCTD16	Plasma Membrane	Other
				1.063	KLF10	Nucleus	Transcription regulator
				–0.613	KRAS	Cytoplasm	Enzyme
				2.749	LEPR	Plasma Membrane	Transmembrane receptor
				–0.69	LIMS1	Plasma Membrane	Other
				1.168	LIPC	Extracellular Space	Enzyme
				0.887	LRP8	Plasma Membrane	Transmembrane receptor
				–1.079	MAGI3	Cytoplasm	Kinase
				–1.167	MAOB	Cytoplasm	Enzyme
				–0.87	MEOX2	Nucleus	Transcription regulator
				–0.634	MGP	Extracellular Space	Other
				1.685	MS4A6A	Other	Other
				–1.838	MSTN	Extracellular Space	Growth factor
				2.404	NEFH	Cytoplasm	Other
				–2.808	NOS2	Cytoplasm	Enzyme
				0.816	NOTCH4	Plasma Membrane	Transcription regulator
				–0.977	NRAS	Plasma Membrane	Enzyme
				1.836	NTRK1	Plasma Membrane	Kinase
				2.42	OLR1	Plasma Membrane	Transmembrane receptor
				–1.059	OPRD1	Plasma Membrane	G-protein coupled receptor
				–1.268	OXCT1	Cytoplasm	Enzyme
				2.329	PAK1	Cytoplasm	Kinase
				0.7	PECAM1	Plasma Membrane	Other
				1.068	PIK3IP1	Cytoplasm	Other
				2.175	PKLR	Cytoplasm	Kinase
				–2.188	PLPP3	Plasma Membrane	Phosphatase
				0.697	POR	Cytoplasm	Enzyme
				–0.754	PPT2	Cytoplasm	Enzyme
				–0.61	PRKCA	Cytoplasm	Kinase
				–0.713	PRKCB	Cytoplasm	Kinase
				0.894	PSMD8	Cytoplasm	Other
				–1.882	PTGIS	Cytoplasm	Enzyme
				–1.791	QPCT	Cytoplasm	Enzyme
				–1.546	RAMP3	Plasma Membrane	Transporter
				–3.845	RET	Plasma Membrane	Kinase
				–0.767	S100A10	Cytoplasm	Other
				1.12	S100B	Cytoplasm	Other
				–0.842	SERPINE2	Extracellular Space	Other
				–0.833	SERPINF1	Extracellular Space	Other
				–0.586	SET	Nucleus	Phosphatase
				2.599	SLC12A1	Plasma Membrane	Transporter
				2.338	SLC12A3	Plasma Membrane	Transporter
				–1.043	SLC2A4	Plasma Membrane	Transporter
				1.018	SLC9A1	Plasma Membrane	Ion channel
				–0.882	SLCO2A1	Plasma Membrane	Transporter
				0.834	SORL1	Cytoplasm	Transporter
				1.193	SOX2	Nucleus	Transcription regulator
				0.936	SQSTM1	Cytoplasm	Transcription regulator
				2.28	SSTR2	Plasma Membrane	G-protein coupled receptor
				0.737	SSTR3	Plasma Membrane	G-protein coupled receptor
				2.067	STAR	Cytoplasm	Transporter
				0.641	TANGO2	Cytoplasm	Other
				1.49	TGFBR3	Plasma Membrane	Kinase
				–2.148	TGM1	Plasma Membrane	Enzyme
				–0.6	THBD	Plasma Membrane	Transmembrane receptor
				0.734	THRB	Nucleus	Ligand-dependent nuclear receptor
				0.828	TIMP3	Extracellular Space	Other
				–3.057	TLR7	Plasma Membrane	Transmembrane receptor
				–0.685	TMEM116	Other	Other
				0.736	TNNT2	Cytoplasm	Other
				–2.696	TOP2A	Nucleus	Enzyme
				–0.705	TP53	Nucleus	Transcription regulator
				2.123	TSPAN5	Plasma Membrane	Other
				–0.83	TUBA8	Cytoplasm	Other
				0.823	XDH	Cytoplasm	Enzyme

Green indicates the downregulated gene; red indicates the upregulated gene.

### Pathway Analysis and Pathophysiological Mechanisms

The contribution of every DEGs to the development of diastolic dysfunction was calculated in the context of molecular network and biological progresses using the algorithms described in the Materials and Methods section.

#### Dahl/SS

**Figure 1:**
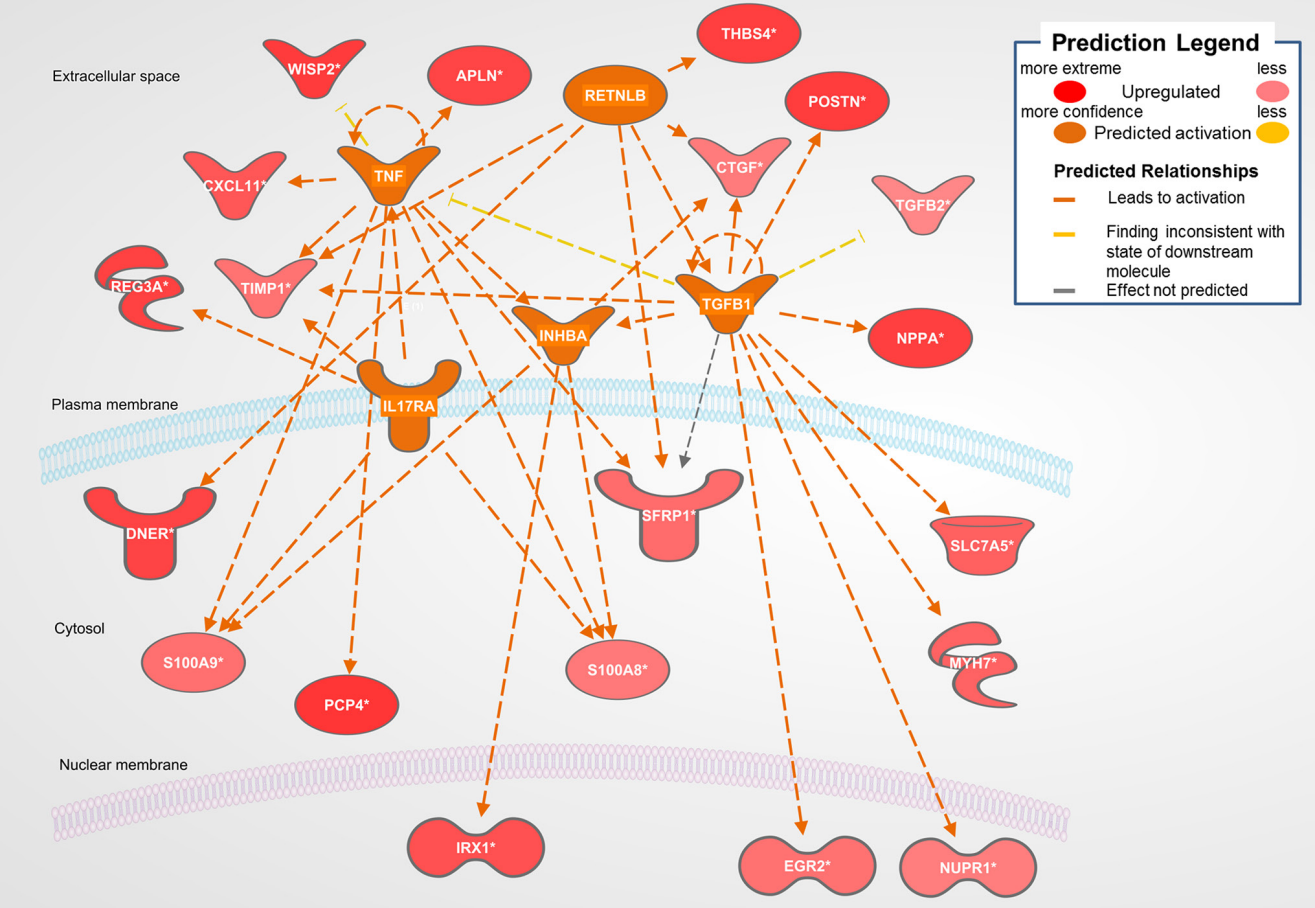
Top predicted activation state in Dahl SS. Asterisks (*) indicate that multiple identifiers in the dataset file map to a single gene in the Global Molecular Network. Figure has been generated using Ingenuity Pathways Analysis (IPA; Ingenuity Systems, Inc., Cambridge, MA, USA).

**Figure 2:**
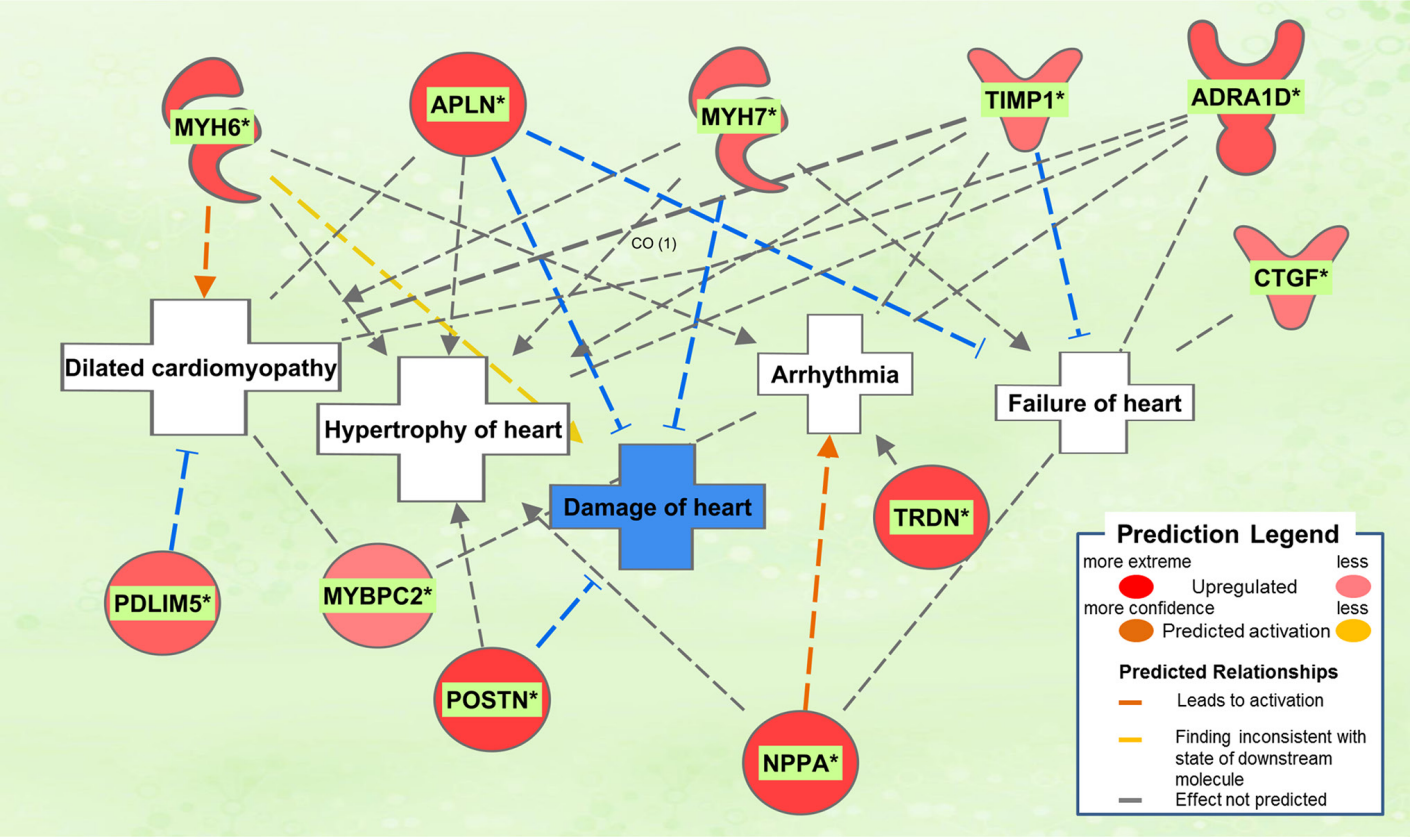
Top pathological functions in Dahl/SS. Asterisks (*) indicate that multiple identifiers in the dataset file map to a single gene in the Global Molecular Network. Figure has been generated using Ingenuity Pathways Analysis (IPA; Ingenuity Systems, Inc., Cambridge, MA, USA).

The biological activities occurring in the heart tissue were identified using the IPA Upstream Regulator analytic on the gene expression changes we observed in the dataset. The analysis inferred the likely activation states of 5 transcription regulators given by our DEG dataset. Hence, based on the comparison of the change of direction of the DEGs (i.e., expression in the Dahl/SS model to control) and what is known from the literature we found that the transcription regulators *TGFB1*, *RETNLB*, *TNF*, *INHBA*, and *IL17RA* (*z*-score 2.4, 2.2, 2.1, 2.0, 2.0, respectively) were predicted to be activated (Figure 1). A further examination of the underlying biological activities prompted by our dataset from the hearts of Dahl/SS rats revealed biological activities such as dilated cardiomyopathy, hypertrophy of heart, arrhythmia, and failure of heart (Figure 2).

#### SHHF

**Figure 3:**
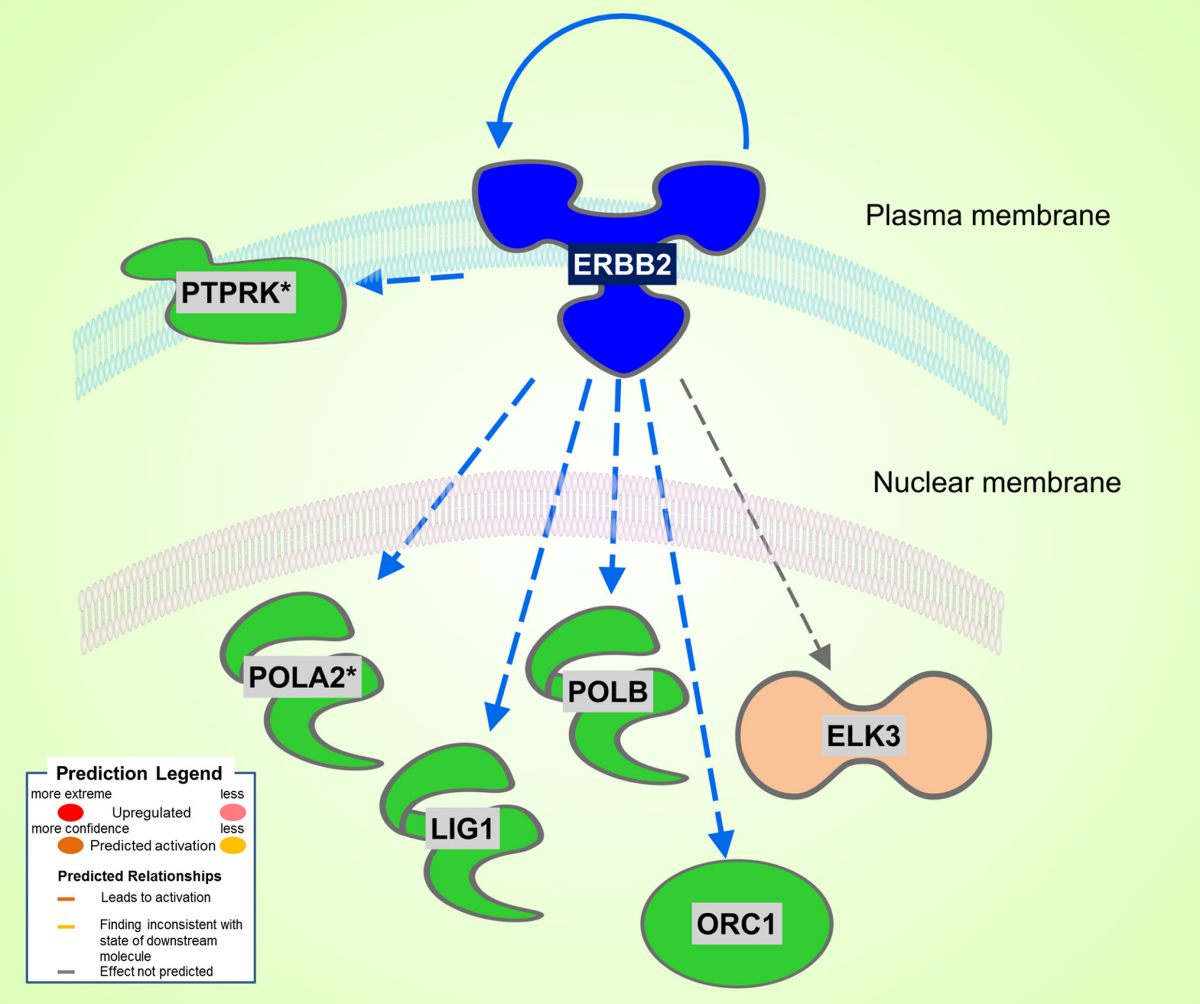
Significant inhibition of ERBB2 kinase in SHHF. Asterisks (*) indicate that multiple identifiers in the dataset file map to a single gene in the Global Molecular Network. Figure has been generated using Ingenuity Pathways Analysis (IPA; Ingenuity Systems, Inc., Cambridge, MA, USA).

A total of 186 DEGs were observed. Of the 78 significantly upregulated genes, 43 fell into the rather nondescript category of “other”, followed by 10 in the second largest group transcription regulator. Of the 108 downregulated genes, 61 were characterized as “other”, 17 as enzyme, and 6 as transcription regulator. Differently from the Dahl/SS, the SHHF model did not show significant inferred activation of upstream transcription regulators, apart from a moderate activation (*z*-score 0.3) of *NUPR1*. In this model, the *ERBB2* upstream regulator was predicted to be inhibited (*z*-score −2.2). As shown in Figure 3, ERBB2 is a plasma membrane-bound receptor tyrosine kinases that is involved in the protection of cardiac myocytes, which is illustrated by the cardiotoxicity of ERBB2-targeted cancer therapies (13). Out of the 250 DEGs determined in the SHHF heart tissue, 26 genes were linked to cardiovascular disease including cardiotoxicity, pericarditis, ST-elevated myocardial infarction, and dilated cardiomyopathy. A list of these genes is provided in Table 3.

#### Diabetic 1

**Figure 4:**
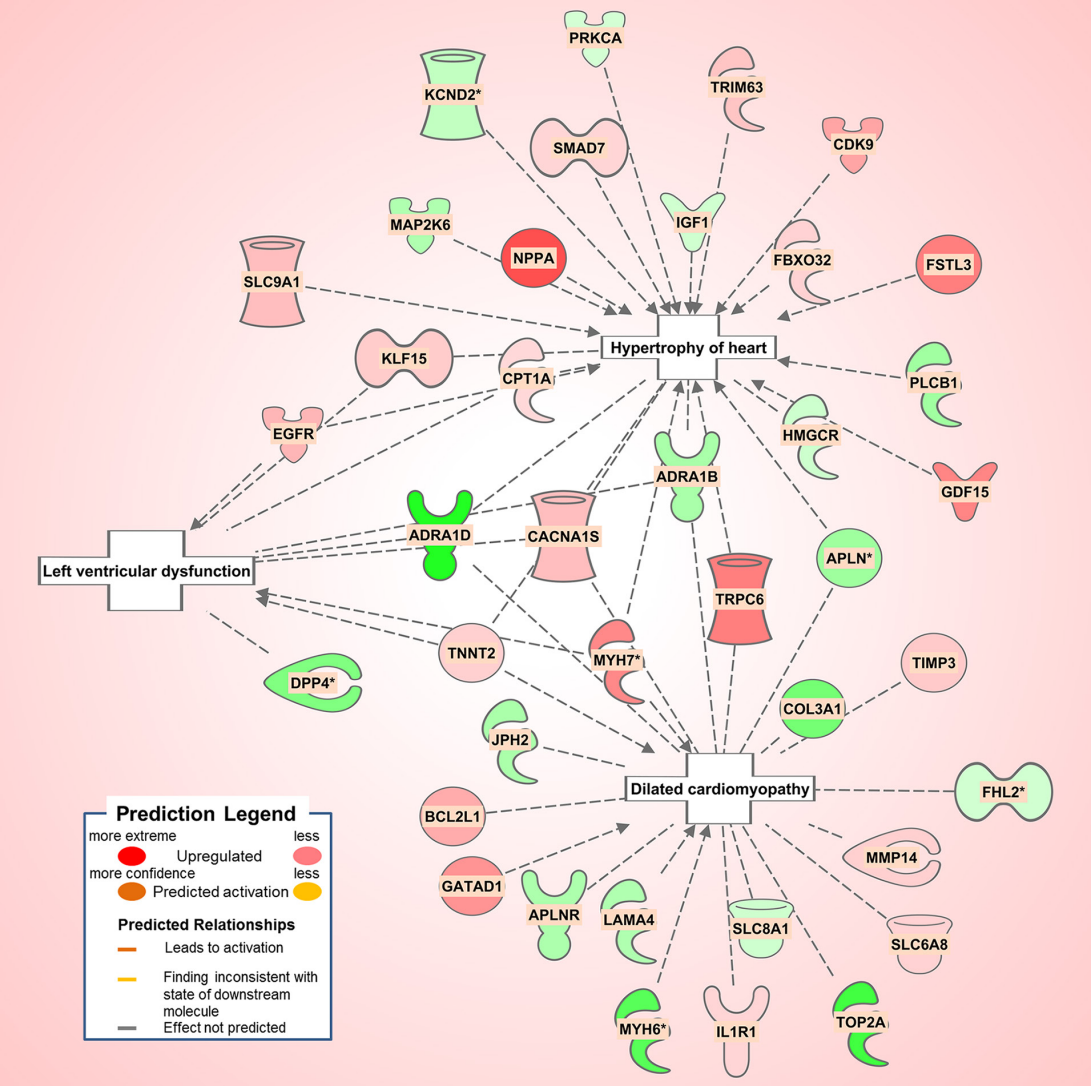
Pathological processes in the diabetic rat model. Asterisks (*) indicate that multiple identifiers in the dataset file mapto a single gene in the Global Molecular Network. Figure has been generated using Ingenuity Pathway Analysis (IPA; Ingenuity Systems, Inc., Cambridge, MA, USA).

The diabetic rat model resulted in the highest number of DEGs (see Table 1) consistent with a FDR = 0. A total of 18 (9 activated and 9 inhibited) significant upstream transcriptional regulators that can explain the observed gene expression profile were identified. A list of these genes is displayed in Table 5. Not surprisingly, given the metabolic underpinnings of the model, most (110) of the 558 genes that were upregulated code for enzymes. Other groupings include 30 kinases, 20 G-protein-coupled receptors, 18 ion channels, and 6 growth factors. Upregulated as well is *NPPA* encoding for atrial natriuretic peptide (ANP), a biomarker for cardiac hypertrophy and heart failure (Figure 4). Two genes that code for recently proposed novel biomarkers for HFpEF were also upregulated: *HSPB1*, which codes for cardioprotective heat shock protein 27 (hsp27) (14, 15) and *ANG* which codes for angiogenin, a protein important for vascularization (16).

**Table 5:** Significant upstream transcriptional regulators in the diabetic rat model

**Upstream Regulator**	**Molecule Type**	**Predicted Activation State**	**Activation z-score**	**Target molecules in dataset**
RABL6	Other	Inhibited	−2,010	ABAT,AURKB,BIK,CCNB1,DAPK1,H2AFX,MCM5,MCM7,RFC3,SERPINH1,TOP2A,TP53
MAX	Transcription regulator	Inhibited	−2,000	CCND2,CDKN2B,FBXO32,GADD45B,KLF10,TMEM126A,TXNIP
HLX	Transcription regulator	Inhibited	−2,000	BMP2,CCNB1,CYP1B1,GDF15
IFNL1	Cytokine	Inhibited	−2,186	EIF2AK2,IFI35,MX1,UBE2L6,USP18
MNT	Transcription regulator	Inhibited	−2,000	FBXO32,GADD45B,KLF10,TXNIP
IFNG	Cytokine	Inhibited	−2,668	ALOX15,ASS1,BCL2L1,CASP8,CAT,Ccl2,CCL22,CYP2E1,DDR2,DPP4,EIF2AK2,ICOSLG/LOC102723996,IFIT1B,LOX,MX1,NOS2,NPY2R,SDC4,SERPINB9,SLC15A3,STAR,TGM1,TXNIP,UBD,USP18
IFNA2	Cytokine	Inhibited	−2,068	EIF2AK2,IFI35,IFNB1,MX1,TP53,UBE2L6,USP18
PCDH11Y	Other	Inhibited	−2,236	CCND1,CCND3,CD44,GJA1,RET
SPDEF	Transcription regulator	Activated	2,496	CDH11,COL1A1,COL4A1,COL4A2,COL5A1,COL5A2,COL6A1,COL6A3,DKK3,EGFR,ITGA6,LAMC1,PRKCA,SMAD3
KIAA1524	Other	Activated	2,359	C4BPA,CDKN2B,COL8A1,ENO3,GPNMB,LUM,RHOC,SERPINE2,TUBA4A
miR-29b-3p (and other miRNAs w/seed AGCACCA)	Mature microrna	Activated	2,449	COL1A2,COL5A2,GAS7,LAMC1,MYBL2,TUBB2A
CST5	Other	Activated	2,584	ACAT2,AKAP12,AP1M2,CAV1,CD44,DECR1,DRAP1,ELK3,EMP3,EPN3,FER,FHL1,LAMC1,NR2F1,NRP1,PLS3,PRSS8,S100A11,TAF1,TGIF1,TSPAN5,VCAN
PTGS2	Enzyme	Activated	2,425	BCL2L1,Ccl2,IFNB1,ITGA6,MCL1,MMP14,PPA1,TP53
SIRT1	Transcription regulator	Activated	2,049	ATXN10,BNIP3,CCND2,CPT1A,CYP1A1,HMGCR,IGF1,TP53
KITLG	Growth factor	Activated	2,000	BCL2L1,IL1RL1,PRKCA,PRKCB
FOXO3	Transcription regulator	Activated	2,197	BNIP3,CAT,CCND2,CDKN2B,EGR4,FBXO32,GADD45B,MXI1,TXNIP
NEDD9	Other	Activated	2,646	BMP2,BNIP3,ELF3,FABP1,GDF15,MMP14,TXNIP
POU2F2	Transcription regulator	Activated	2,236	ALDOC,KCNN4,MCM7,PFKFB3,SSTR2,VIP

Orange/blue indicate the predicted activation state, namely activated/inhibited, of the transcriptional regulator.

## Discussion

In our study, we selected three established rat models of diastolic dysfunction that are defined by three major risk factors associated with HFpEF. Remarkably, we found that the cardiac transcriptomics of the models demonstrated little commonality, either in differentially regulated genes or specific biological functions. Overall, our findings highlight the case that divergent molecular processes may underpin a shared phenotype.

Diastolic dysfunction is primarily characterized by stiffening of the left ventricular (LV) tissue, which contributes to impaired relaxation and filling of the ventricle. The specific mechanisms leading to myocardial stiffening are undetermined, but broadly LV stiffness has active and passive components. Passive stiffness describes the inherent stretchiness of the myocardium and is governed by the pliability of both the extracellular matrix and the contractile units. The latter is set by the giant sarcomeric filament, titin, which also determines the elastic recoil of the myocardium, thereby contributing in a major way to LV filling *via* diastolic suction (17). Evidence indicates that abnormal high-energy phosphate metabolism, specifically increased free cytosolic ADP and enhanced AMP catabolism, may contribute to passive myocardial diastolic stiffness as well (18, 19).

Active stiffness, or reduced relaxation, of the LV tissue depends on Ca^2+^ homeostasis (20). There are two interrelated aspects of intracellular Ca^2+^ handling that determine diastolic function; the rate of cytosolic Ca^2+^ decline following its release from the sarcoplasmic reticulum (SR), and the resting or end-diastolic Ca^2+^ concentration (21). These parameters are set by the rate of Ca^2+^ removal from the cell *via* the sodium calcium exchanger (NCX) and reuptake of Ca^2+^ into stores by SERCA2, in addition to Ca^2+^ leak from the SR. We did not observe any changes in the gene expression of calcium handling proteins (SERCA2, NCX, PLN, or CASQ2) in any of the three models of diastolic dysfunction. Of course, our approach does not address the possibility that in any of the models of diastolic dysfunction the activities of the Ca^2+^ handling protein are negatively affected by posttranslational modification or change in their environment.

Figure 1 displays the targets of TGFB1 that are differentially expressed in the Dahl/SS dataset. TGFB1 plays a central role in hypertension-induced cardiac hypertrophy and fibrosis (22, 23), and consistent with that fact, genes involved in fibrosis/extracellular matrix (ECM) accumulation (*TGFB1*, *POSTN*, *CTGF*, *TIMP1*) (24) and hypertension-related pathological hypertrophy (*NPPA* and *MYH7*) (25–28) are upregulated with hypertension. Interestingly, the Dahl/SS model of heart failure is associated with upregulation of the Wnt signaling inhibitor secreted frizzled-related protein-1 (*SFRP1*), which protects against ventricular dilation and hypertrophy (29), as well as increased expression of the transporter of large neutral amino acids (*SLC7A5*). Downstream targets of TGFB1 upregulated in Dahl/SS rats include *EGR2* and *NUPR1*. Based on computational analysis of the transcriptomes at five time points in male murine hearts subjected to transverse aorta banding, the transcription factor EGR2 was recently identified as a new candidate key regulator of cardiovascular-associated genes (30). Nupr1 is a nuclear basic helix-loop-helix protein and transcriptional coregulator that is induced in response to stress in the heart (31). Recent evidence indicates that Nupr1 activation contributes to cardiac fibrosis, while protecting against autophagy and apoptosis that lead to heart failure (32). Elevated circulating levels of both interleukin (IL)-6 and TNF are observed in HFpEF (33), and in the Dahl/SS model TNF shares several downstream targets with TGFB1, including *TIMP1*, *INHBA*, and *SFRP1* (Figure 1). The role of INHBA or RETNLB in cardiac remodeling is unreported. The predicted downstream target of RETNLB, THBS4, has been shown to attenuate pressure-overload cardiac fibrosis (34). TNF is also linked to *S100A9* and *S100A8* expression (both significantly increased in our DEG list), as is *IL17RA* (Interleukin 17 receptor A). IL17RA has been linked to myocardial collagen metabolism in hypertension-induced diastolic dysfunction and heart failure (35, 36). The “damage-associated molecular patterns” (DAMPs), proteins S100A8/A9 contribute to cardiac fibrosis by activating pro-inflammatory NF-κB signaling in cardiac fibroblasts *via* the receptor for advanced glycation end products (RAGE) and inducing the expression of multiple chemokines and cytokines (37). NF-κB is predicted to be activated in the Dahl/SS model (z-score of 1.92), as evidenced by the marked upregulation of *CTGF*, *CXCL11*, *SLC7A5*, *TIMP1*, and *CDC25B*. TNF is also linked to upregulation of the chemokine CXCL11 and the hormone Apelin, which appear to have conflicting actions on cardiac inflammation (38) and hypertrophy (39), respectively. Interestingly, TNF is linked to upregulation of Purkinje cell protein-4 (*PCP4*), a putative regulator of calmodulin and Ca^2+^/calmodulin-dependent kinase II (CaMKII) signaling, within the His-Purkinje network and contributor to cardiac arrhythmias (40). The role if any played by *DNER*, *REG3A*, and *IRX1* to diastolic dysfunction and cardiac hypertrophy is not reported, although genetic variants of the *IRX1* gene were recently reported to contribute to the pathogenesis of congenital heart disease (41). Finally, expression levels of CXCL11 were increased in hearts of Dahl/SS rats fed a high salt diet, which is consistent with the concept that inflammation plays a key role in the pathogenesis of HFpEF. The closely related CXCL9, CXCL10, and CXCL11 are chemoattractants for monocytes and lymphocytes. Accumulating evidence has implicated these chemokines in several cardiovascular diseases, including atherosclerosis, hypertension, cardiac hypertrophy, and heart failure, as well as in transplant coronary artery disease and heart transplant rejection (42). Of note, circulating levels of these chemokines were found to provide additional diagnostic utility beyond NT-pro BNP levels for detecting LV diastolic dysfunction in hypertensive patients (43). In addition, we observed that serum levels of CXCL10 are increased in patients with symptomatic heart failure (44). Although CXCL9, -10, and -11 all bind to CXCR3, there is some evidence that these agonists activate opposing responses due to biased signaling that is a fixture of G protein-coupled receptors (44). Whereas CXCL9/CXCL10/CXCR3 interactions drive effector Th1 polarization, CXCL11/CXCR3 binding seems to induce an immunotolerizing state characterized by polarization into regulatory Tr1 lymphocytes that produce anti-inflammatory IL-10.

For the SHHF dataset, none of the DEGs stood out mechanistically in the context of heart failure. Expression of the gene for frizzled class receptor 5 (*FZD5*), which plays a role in endothelial cells in angiogenesis (45), was increased, as was the gene for the repressor of the protective, anti-oxidant transcription factor Nrf2, *KEAP1* (46).

Unexpectedly, the genes for 9 different collagen types were downregulated in diabetic 1 hearts, although a number of genes involved in ECM remodeling and fibrosis were upregulated, including *MMP14* (24), as well as *SDC4* and *LOX*, which together have been linked to myocardial stiffness and diastolic dysfunction (47). *PDGFD*, which codes for platelet-derived growth factor D, a potent pro-fibrotic cytokine (48), was upregulated. Two signaling components of the pro-fibrotic cytokine TGF-β were upregulated, *SMAD3* (49) and *TGFBR3* (TGF-β receptor 3), although *TGFBR1* (TGF-β receptor 1) was downregulated and the inhibitory *SMAD7* was upregulated. However, *CAV*1 was downregulated. *CAV1* encodes for caveolin 1, which may inhibit both TGF-β-dependent and –independent cardiac fibrosis (50). Other relevant pro-fibrotic genes that were upregulated, include: the chemokines *CCL2* and *CXCL3*, linked to fibrosis and inflammation (51–53); pro-inflammatory cytokine *Il-3* (54) and the innate immune cytokine *IFNB1*; and the K^+^ channel *KCNN4*, which is linked to angiotensin II-induced cardiac fibroblast proliferation and collagen production (55). The gene for the tyrosine kinase receptor EGFR was also upregulated. EGFR is implicated in cardiac fibrosis and hypertrophy (56–58), as well as heart protection (59). In contrast, the gene for TIMP3, which has anti-fibrotic actions (60), was upregulated. Several pro-fibrotic genes were downregulated, including: *VCAN* (Versican) (61); *LUM* (Lumican), an ECM-localized proteoglycan that binds collagen and is important for fibrosis, is associated with inflammation, and is increased in experimental and clinical heart failure (62); *SERPINE2*, which is increased in pressure overload- and angiotensin II-induced cardiac remodeling downstream of ERK1/2 signaling and increases collagen deposition (63); and the integrin *ITGA6*, associated with collagen deposition (64).

Notable genes linked to metabolism were increased in diabetic hearts, including *FABP1* and *GDF15*. The later encodes a heart-derived hormone, which regulates body growth (65). *PFKFB3* encodes a pro-glycolytic enzyme that is upregulated in cardiac progenitor cells by diabetes (66). The gene for the leptin receptor (*LEPR*), which is implicated to both cardiac hypertrophy and cardioprotection in obesity (67), was upregulated. However, a number of genes associated with cardiac hypertrophy and heart failure due to pressure-overload or angiotensin II infusion were downregulated. *CCND2* (cyclin D2), which was implicated in cardiac hypertrophy, was downregulated (68), but so was the cyclin-dependent kinase inhibitor *CDKN2B. TP53*, a major player in the development of systolic heart failure (69, 70), was downregulated, as was *PRKCA*. Activation of PKCα is implicated in cardiac hypertrophy and heart failure (71–73).

Oxidative stress is a feature of the diabetic heart (74) and several genes associated with inflammation and oxidative stress were upregulated. *CAT*, encoding the key antioxidant enzyme catalase was upregulated. On the other hand, *TXNIP*, which inhibits the anti-oxidative function of thioredoxin, was also upregulated. TXNIP is linked to increased oxidative stress, along with fibrosis and arrhythmias in diabetes (75), as well as diabetic cardiomyopathy (76). The thioredoxin system 2 is a major buffer against H_2_O_2_ emission from mitochondria (77), and the present evidence showing that *TXNIP* is strongly upregulated in the diabetic heart may account for the overall oxidative environment found in the diabetic heart.* TSPAN5*, encoding a cell surface protein was upregulated. Evidence was recently reported that endothelial Tspan5- and Tspan17-ADAM10 complexes contribute to inflammation by maintaining VE-cadherin expression and promoting T lymphocyte transmigration (78). Another injury-related gene that was upregulated was *BNIP3*, which is associated with mitochondrial autophagy and apoptosis and is increased in the heart by pressure overload and various stresses (79, 80).

Notably, a number of protective genes were upregulated in the type 1 diabetic heart. The gene for the Smad corepressor, transforming growth interacting factor (*TGIF*) was increased, as was *BMP2*. BMP2 may antagonize BMP4-induced cardiomyocyte hypertrophy and apoptosis (81) and TGF-β-induced fibrosis (82). Three genes linked to inhibition of cardiac hypertrophy were upregulate, *viz.*, *DKK3* (Dickkopf-3) (83), *FBXO32* (encoding E3 ligase, Fbxo32) (84), and *GADD45B*, which blocks MKK7-induced JNK activation (85). The anti-apoptotic gene *BCL2L1* (86) was upregulated, as well as *MCL1*, which is important in mitochondrial function and autophagy (87). *VIP* (vasoactive intestinal peptide), cardioprotective in heart failure (88), and *SMAD5*, which has anti-apoptotic actions in cardiac myocytes (89) were upregulated. On the other hand, *AKAP12*, which may protect against IR injury (90), was downregulated, as were both *MYBL2* and *TUBB2A*, which were recently shown to be upregulated in the heart after acute MI (91, 92).

Interestingly, we found that there were no genes similarly affected among all three models of diastolic dysfunction, nor between the Dahl SS and SHHF models. However, the type 1 diabetes model shared 20 regulated genes with the Dahl SS model and 15 with the SHHF model. Human diseases traditionally have been differentiated and categorized on the basis of which organ system they primarily affect. An alternate view is now emerging, which emphasizes that different diseases typically have common underlying mechanisms and intermediate pathophenotypes or endo(pheno)types. According to this construct, expression of a specific disease reflects the interplay between relevant endophenotypes and the local organ-based environment. Important examples of such endophenotypes are inflammation, fibrosis, and metabolic dysfunction, which play essential roles in many developing diseases. In this study, we identified the endophenotype networks of three models of diastolic dysfunction and explored their relation to cardiovascular diseases in particular. We identified the subnetworks of the top regulated microarray genes that are playing a role in inflammation, fibrosis, and metabolism. Although our three models of diastolic dysfunction exhibit little overlap in regulated genes, each is still significantly enriched with disease-associated genes. Moreover, they are enriched also with differentially expressed genes linked to cardiovascular disease (risk).

Our findings illustrate as well the observation that HFpEF is a syndrome with multiple extracardiac comorbidities, such as aging, hypertension, and obesity, rather than a disease of clearly defined etiology (93). Pharmacological targeting of HFpEF may need to be informed more by phenotypic characteristics of the cardiac remodeling events that occur than by the underlying molecular processes alone. These phenotypic events may include concentric hypertrophy, myocardial stiffness, fibrosis, endothelial dysfunction, capillary rarefaction, and abnormal ventricular-arterial coupling, as well as changes in the shape and stiffness of the left atrium.

There are some limitations and caveats to our approach. Changes in gene expression may not necessarily be reflective of changes in protein levels, given the contribution of additional levels of post-transcriptional regulation, including microRNA mediated regulation. Alterations in protein stability and function may occur as well. In addition, in any disease context, changes in gene expression have a temporal and dynamic component, with alterations in expression of any particular gene and its protein levels at a given time point being impacted upon by changes in other network or system components. Nonetheless, global changes in gene expression are reflective of basic underlying pathological processes and offer insight into stress-related responsiveness to the disease process. In addition, there are several other models of HFpEF, such as abdominal aortic constriction and partial nephrectomy. Certainly, it would be of interest to extend our analysis in the future to include these other models. It should be mentioned as well that streptozotocin affects other organ systems, including vascular function; however, HFpEF in many patients is increasingly recognized as a systemic disorder involving vascular inflammation and dysfunction (94).

In conclusion, our *in silico* analysis of differential gene expression in three common rat models of diastolic dysfunction highlights the diversity in causality and molecular basis for the impaired cardiac function. A better understanding of the phenotype changes that accompany diastolic dysfunction, such as concentric hypertrophy, and their impact on genetic changes, is required to gain better insight into the disease process. An effective pharmacological approach for diastolic dysfunction and HFpEF may arise from a global strategy based on a better understanding of altered cardiac ultrastructure.

## Author Contributions

RA conceived the study, analyzed the data, designed the figures and tables, and helped write the manuscript. FZ and RB assisted in the analysis of the data and writing of the manuscript. SB and AC helped write the manuscript. GB assisted in the analysis and interpretation of the results, and contributed to the writing and editing of the manuscript.

## Conflict of Interest Statement

The authors declare that the research was conducted in the absence of any commercial or financial relationships that could be construed as a potential conflict of interest.

The reviewer VO and handling Editor declared their shared affiliation.
