# Supplementary figures and images for: In Silico Analysis of Differential Gene Expression in Three Common Rat Models of Diastolic Dysfunction
Source: Front Cardiovasc Med. 2018 Feb 21;5:11. doi: 10.3389/fcvm.2018.00011 (PMC5850854; doi:10.3389/fcvm.2018.00011)

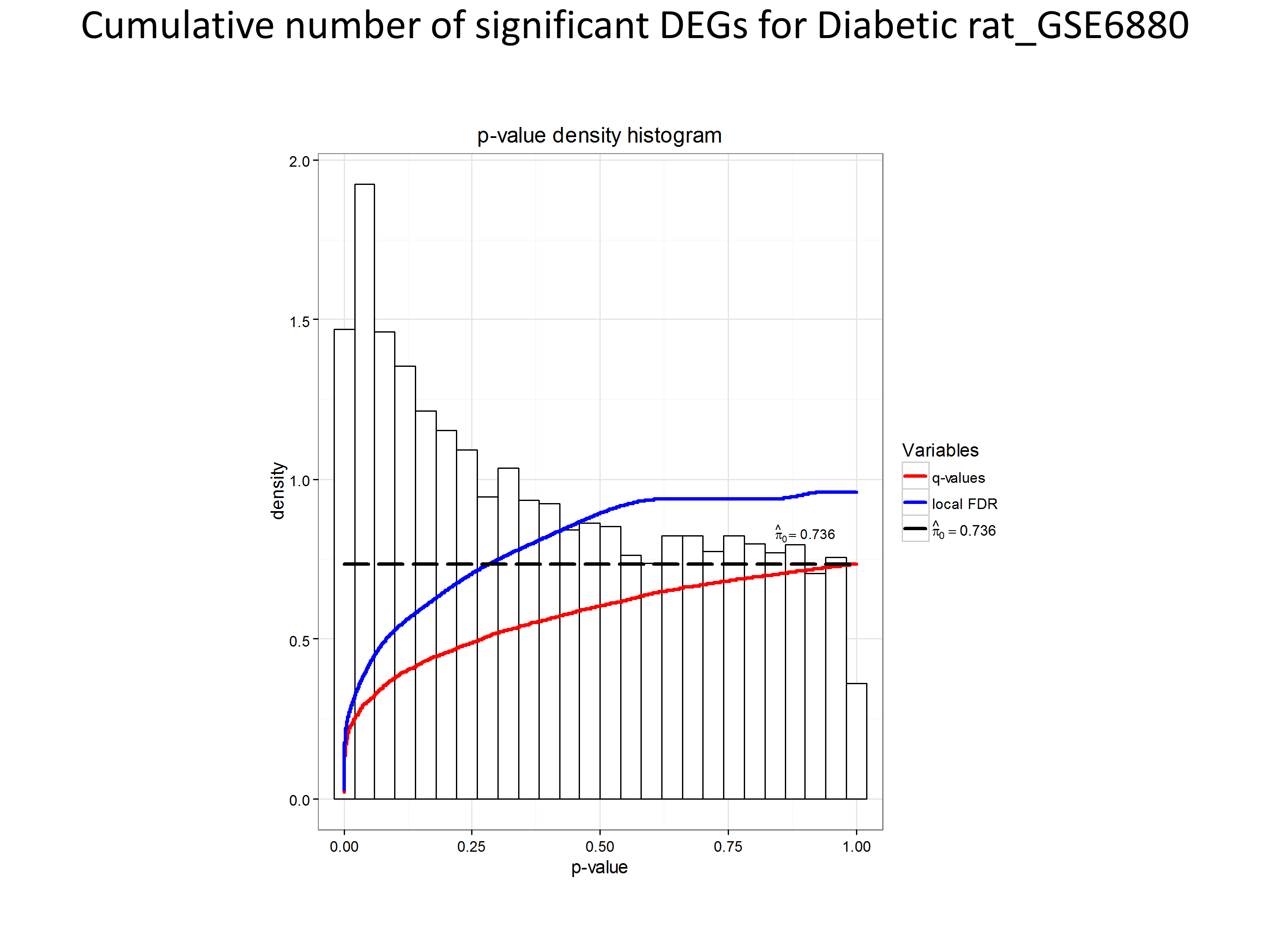

Supplement: Supplementary file 1 [file fcvm-05-00011-s001.tif]
